# Supplementary material for: Aperiodic and oscillatory systems underpinning human domain-general cognition
Source: Commun Biol. 2024 Dec 18;7:1643. doi: 10.1038/s42003-024-07397-7 (PMC11655660; doi:10.1038/s42003-024-07397-7)
Supplement: Supplementary file 2 — Supplementary Information [file 42003_2024_7397_MOESM2_ESM.pdf]

Supplementary material

Table S1 Descriptive statistics: Mean and standard deviation (in brackets) of response error and response time (RT), N = 43

		WM		SWIT		MSIT	
		Easy	Hard	Easy	Hard	Easy	Hard
Accuracy (%)	Alphanumeric	96.48 (4.99)	91.15 (8.63)	96.81 (2.94)	95.74 (3.38)	99.84 (0.54)	96.71 (3.46)
	Colour	96.22 (4.65)	84.98 (6.45)	96.82 (2.86)	95.65 (4.01)	99.64 (0.81)	97.38 (2.29)
RT (ms)	Alphanumeric	652 (111)	769 (135)	1032 (169)	1173 (200)	581 (103)	799 (148)
	Colour	603 (113)	732 (139)	1007 (172)	1170 (205)	615 (123)	828 (158)

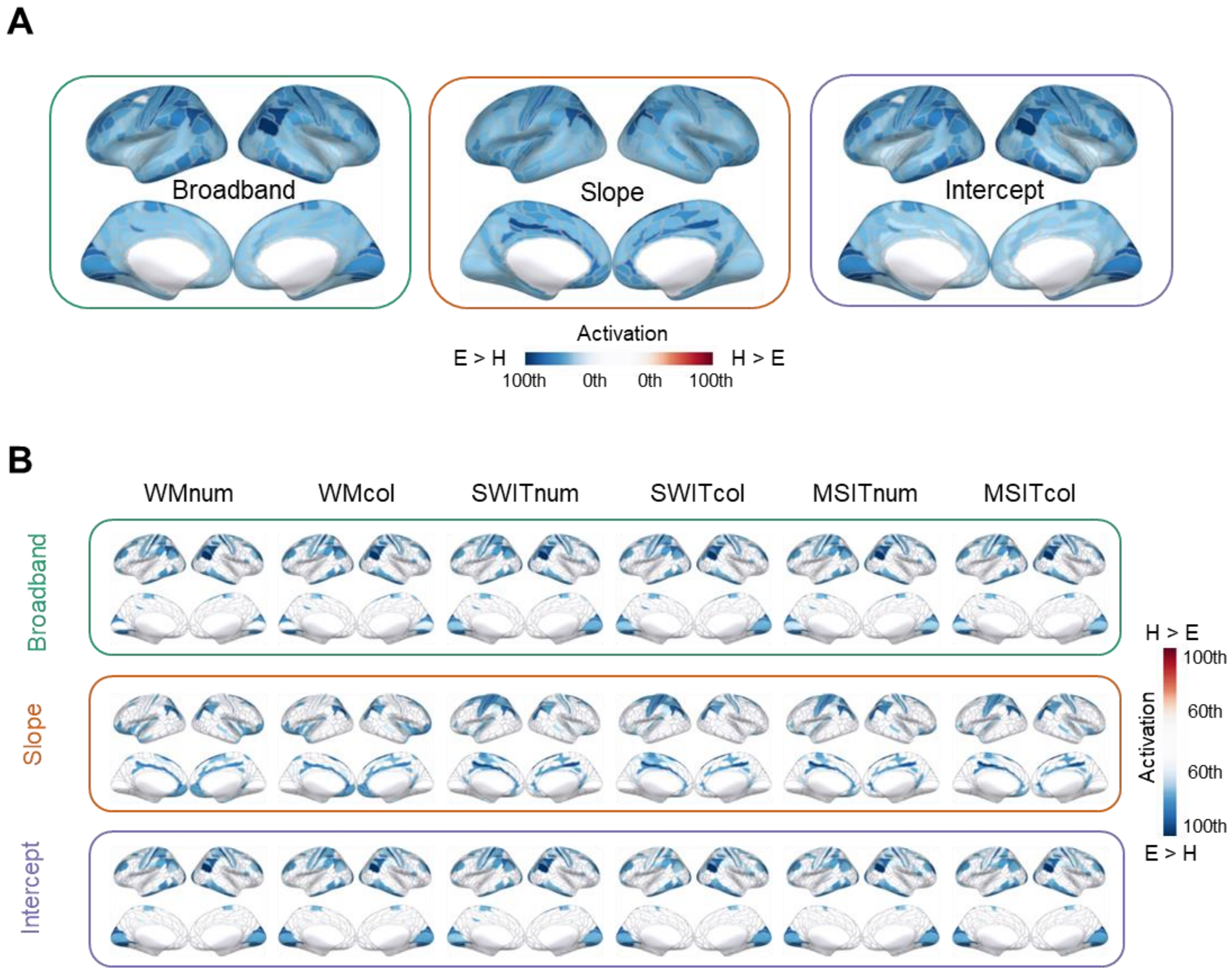

Figure S1. (A) The full map of source estimation patterns for demand decoding (hard vs. easy) averaged across all the subtasks for aperiodic signals. Outlines show 360 cortical regions based on the Human Connectome Project multimodal parcellation (HCP-MMP1.0) [53]. Coloured regions represent the 0th to 100th percentiles of activation across the brain (H: hard; E: easy). (B) Source estimation patterns for demand decoding (hard vs. easy) in each subtask for the aperiodic components. Coloured regions represent the 60th to 100th percentiles of activation across the brain. Abbreviations as shown in Figure 2.

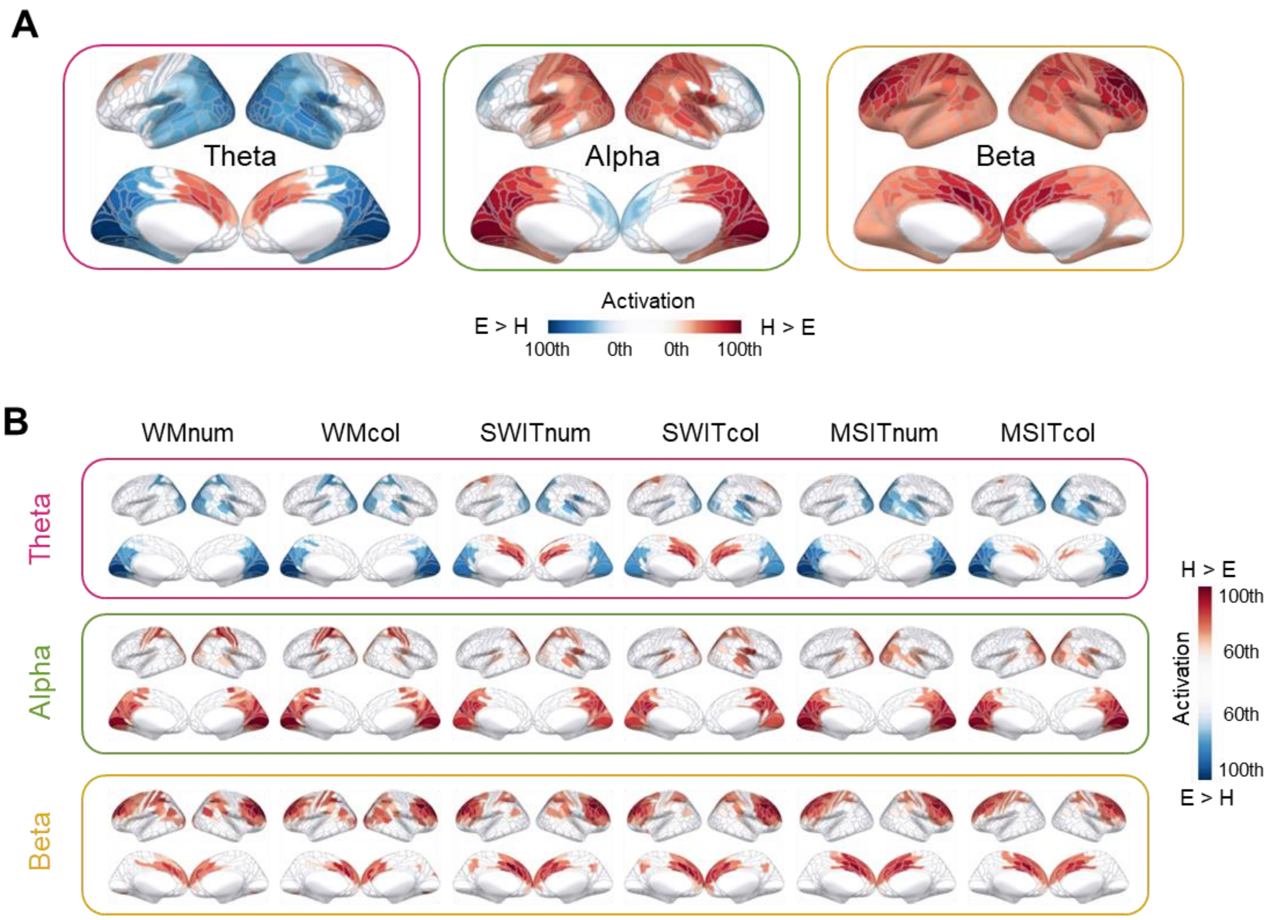

Figure S2. (A) The full map of source estimation patterns for demand decoding (hard vs. easy) averaged across all the subtasks for oscillatory signals. Coloured regions represent the 0th to 100th percentiles of activation across the brain (H: hard; E: easy). (B) Source estimation patterns for demand decoding (hard vs. easy) in each subtask for the oscillatory components. Coloured regions represent the 60th to 100th percentiles of activation across the brain. Abbreviations as shown in Figure 2.

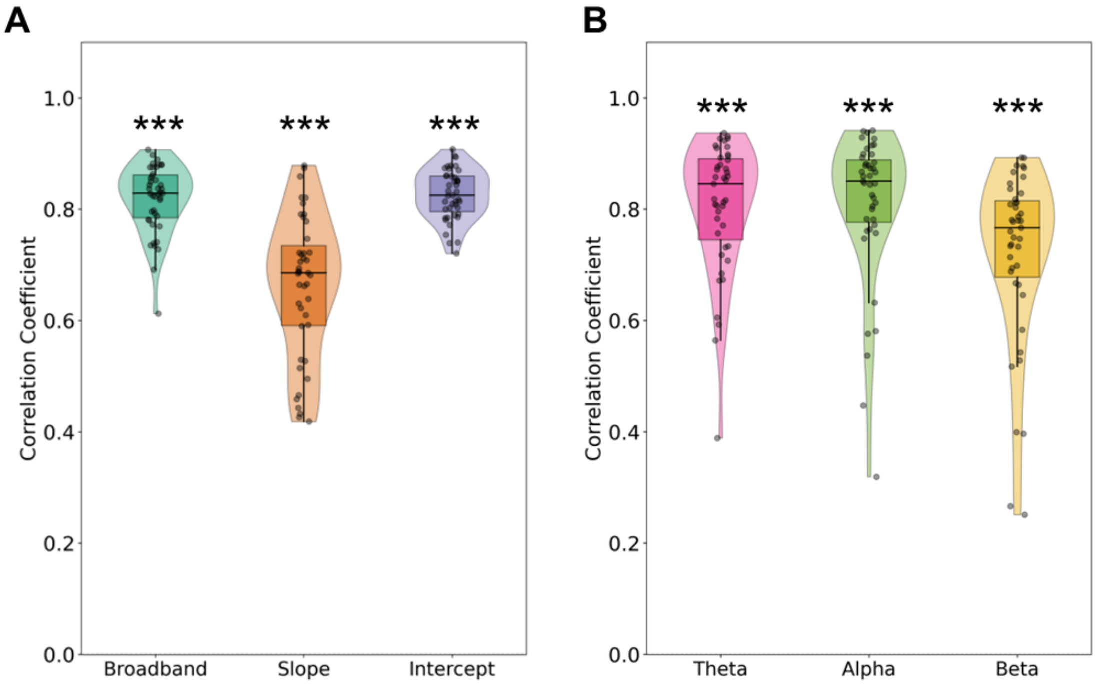

Figure S3. Pearson's correlation results between source estimation patterns that coded demand and content from (A) aperiodic and (B) oscillatory activity across 360 cortical regions in source space. All the signals were averaged across subtasks per region before calculating the correlations. Each dot represents a single participant. *** $p < 0.001$ with 1000 permutations.

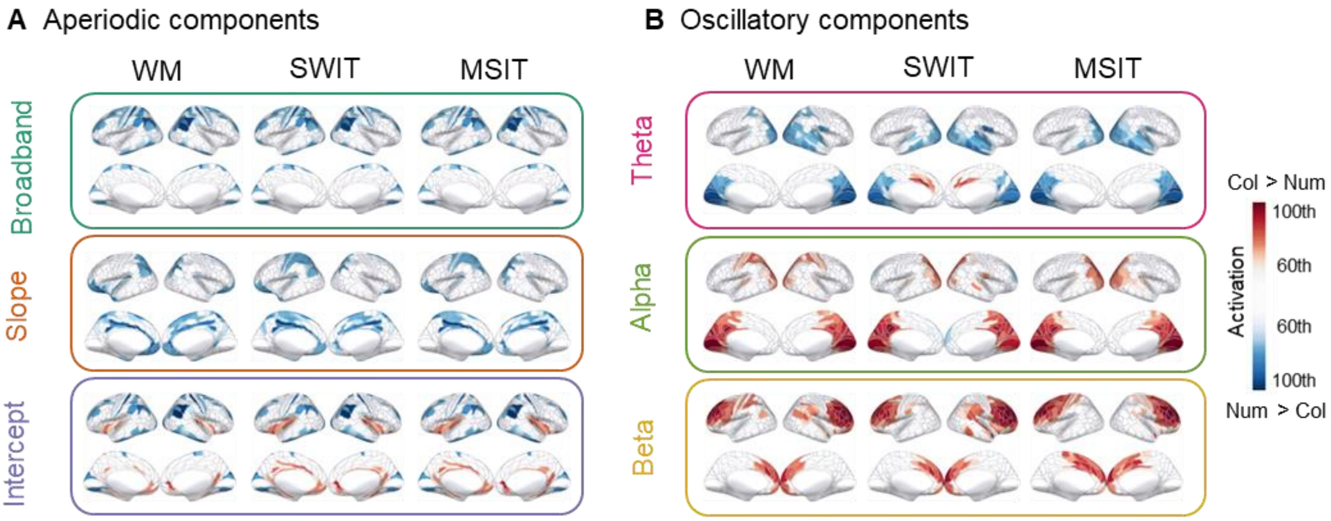

Figure S4. Source estimation patterns for task content decoding (alphanumeric vs. colour) for each task based on (A) the aperiodic and (B) the oscillatory activity. Coloured regions represent the 60th to 100th percentiles of activation across the brain. Abbreviations as shown in Figure 2.

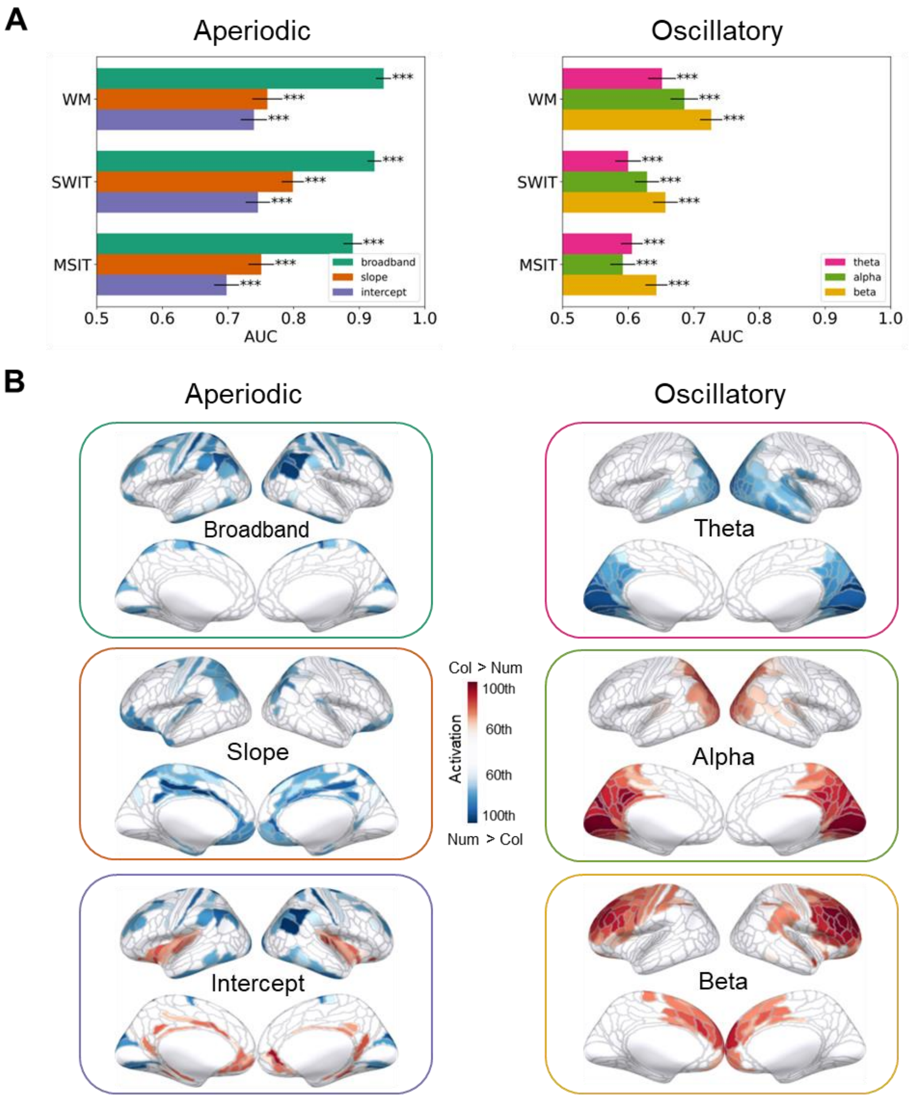

Figure S5. (A) Decoding results on task content (alphanumeric vs. colour) using easy trials only based on the aperiodic and oscillatory activity. (B) Source estimation patterns averaged across all the tasks using easy trials only based on the aperiodic and oscillatory activity. Coloured regions represent the 60th to 100th percentiles of activation across the brain. Abbreviations as shown in Figure 2.
